# Effects of Vapor Gard (Di-1-*p*-menthene) on Potato (*Solanum tuberosum* L.) Yield and Tuber Physiological Disorders Under Moderate and Severe Drought

**DOI:** 10.3390/plants15040536

**Published:** 2026-02-09

**Authors:** Oluwatoyin Favour Olu-Olusegun, Aidan Farrell, James Monaghan, Peter Kettlewell

**Affiliations:** 1 Crop Science Group, Harper Adams University, Newport TF10 8NB, Shropshire, UK; jmonaghan@harper-adams.ac.uk; 2 Department of Life Sciences, The University of the West Indies, St. Augustine Campus, St. Augustine, Trinidad and Tobago; aidan.farrell@uwi.edu

**Keywords:** relative water content, stomatal conductance, physiological disorders, water stress mitigation

## Abstract

Potatoes (*Solanum tuberosum* L.) are highly sensitive to water deficits, which compromise both yield and tuber quality, with russeting representing a major disorder that limits marketability. Film-forming antitranspirants, such as Vapor Gard (VG; di-1-*p*-menthene), may alleviate these effects by reducing transpirational water loss, but their effectiveness under different stress intensities remains uncertain. This study investigated the impacts of moderate and severe drought (targeted at 30% and 20% available water content, respectively) on plant water status, yield, and tuber physiological disorders in two varieties (Challenger and Russet Burbank), and assessed the extent to which VG could mitigate these impacts. Two pot experiments were conducted in a polytunnel using a factorial combination of soil–water regime and VG application. Drought reduced relative water content (RWC) by 30–36% and tuber yield by 29–61%, compared with irrigated plants. Under moderate drought, VG improved leaf RWC by 14–27% and increased yield by 37–67% relative to untreated drought-stressed plants across the two experiments, approaching levels achieved with irrigation. VG’s influence on stomatal conductance was small and inconsistent. VG also consistently reduced russeting in the susceptible Challenger variety, while reductions in necrosis and jelly end rot were observed in Russet Burbank, indicating that disorder responses were variety dependent. Under severe drought conditions, VG provided little additional benefit for plant water status, yield, or disorder incidence. Overall, the results suggest that VG has potential as a management tool for reducing drought-related yield and quality losses in potatoes, particularly under moderate soil water deficit. However, further optimisation of application strategies is required to enhance consistency across environments.

## 1. Introduction

Potatoes (*Solanum tuberosum* L.) are a globally important staple crop, ranking fourth in worldwide production [1]. They play a vital role in food security and economic resilience, particularly in developing regions [2]. However, climate change, especially rising temperatures and prolonged drought, poses a serious threat to potato production. Yield losses due to drought can reach up to 50% [3], with estimates indicating a 2% yield reduction for every 10% decline in rainfall under non-irrigated conditions [4].

Potatoes’ shallow root systems and high-water demands make them highly susceptible to drought stress [5]. Drought impairs key physiological functions, leading to reduced growth, lower yields, and increased vulnerability to disease [6]. Additionally, water deficits can diminish tuber quality and storage potential by promoting physiological disorders such as russeting, internal browning, and bruising [7,8,9], which can worsen during storage [10] and contribute to post-harvest losses.

One potential strategy to mitigate the effects of drought on crops is the use of antitranspirant substances, which reduce water loss by limiting transpiration. These compounds help maintain plant water status by reducing stomatal conductance (SC) and preserving leaf water potential [11]. In potatoes, AT application under controlled conditions has reduced transpiration by 38–50% [12], increased total and marketable yield by 20% and 1.7%, respectively [13], and enhanced photosynthetic activity under drought [14]. Field research has similarly reported increased plant height, biomass, and yield under water stress [15].

Film-forming antitranspirants such as Vapor Gard (VG) form semi-permeable barriers on the leaf surface, offering prolonged protection (up to 25 days) while allowing limited gas exchange [16]. VG’s active ingredient, di-1-p-menthene (pinolene), is a natural terpene polymer derived from plant resin. When applied as a foliar water emulsion, it polymerises under sunlight to form a thin, protective film on the leaf surface, reducing transpiration while remaining environmentally safe [17,18]. Recently, we have shown that VG has the potential to improve water retention and reduce the incidence of tuber physiological disorders, such as necrosis and russeting [19].

Despite these benefits, research on the use of antitranspirants in potatoes remains limited. Some studies in potatoes have reported positive effects under drought, including improved yield in specific experiments and reduced russeting, but responses are not always consistent across seasons or cultivars [19]. In other crops, such as wheat, film-forming antitranspirants have shown context-dependent effects, with effectiveness varying by antitranspirant type, application method, and cultivar [20]. Notably, a significant knowledge gap persists regarding whether antitranspirants can directly reduce the incidence or severity of tuber physiological disorders under drought conditions. This gap is critical, as such disorders directly influence tuber quality, storability, and economic value. Furthermore, most studies have focused on yield or physiological parameters, with comparatively fewer investigating quality-related traits under drought stress.

Our previous research demonstrated that VG reduced russeting and improved tuber appearance in drought-stressed Challenger potatoes [19]. However, the results varied between experiments, necessitating further validation. To address this gap, the present study investigates the repeatability and robustness of VG’s effects under more severe drought conditions and broader environmental contexts, including contrasting temperature and radiation regimes. This study aimed to determine whether VG application can mitigate the negative effects of moderate and severe soil drought on potato yield and tuber physiological disorders by influencing plant water status. Specifically, the study assessed the effects of VG on leaf relative water content, stomatal conductance, yield components, and the incidence of key tuber physiological disorders under contrasting drought intensities. The following null hypotheses were tested. (1) There are no differences between moderate drought (DT) and severe drought (DT2) in plant water status, yield, and tuber physiological disorder incidence. (2) VG does not alter relative water content (RWC), stomatal conductance (SC), yield, or disorder incidence under drought stress. (3) There are no differences between VG application under moderate (VGDT) and severe drought (VGDT2) conditions.

By systematically testing these null hypotheses, this study explores whether VG provides consistent benefits to potato drought resilience, yield stability, and tuber quality, and identifies the environmental and varietal conditions under which its effectiveness is maximised.

## 2. Materials and Methods

### 2.1. Research Environment and Planting Materials

Two pot experiments were carried out in 2024 at Harper Adams University (HAU), Newport, Shropshire, UK (52°46′ N, 2°25′ W), within a naturally ventilated polytunnel that provided semi-controlled conditions for drought simulation. Experiment 1 (Exp 1) was established on 12 April 2024, and Experiment 2 (Exp 2) on 24 July 2024. Plastic containers (capacity ~40 L; 50 cm diameter, 36 cm depth) were used, each filled with ~20 kg of John Innes No. 2 compost (LBS Worldwide Ltd., Colne, Lancashire, UK). This commercial mix comprises loam, peat, coarse sand, and a balanced base fertiliser. At planting, the compost water content was adjusted to ~40% volumetric water content (VWC), equivalent to ~95% field capacity, to standardise initial conditions.

Two potato varieties were selected based on their commercial relevance and differing susceptibility to physiological disorders, rather than inherent differences in drought tolerance. Challenger, a processing type sensitive to russeting (HZPC, Scunthorpe, UK) and Russet Burbank, prone to internal defects such as jelly end rot (McCain Ltd., Montrose, Scotland, UK), were used [19]. One certified seed tuber was planted per pot. The antitranspirant tested was Vapor Gard (VG).

### 2.2. Experimental Layout and Treatment Structure

To account for spatial variability within the polytunnel, both experiments were arranged using a randomised complete block design. The treatment structure followed a 2 × 5 factorial, combining two commercial potato cultivars (Challenger and Russet Burbank) with five irrigation/antitranspirant treatments. The treatments arranged in order of increasing drought severity and inclusion of the antitranspirant were:

1. IRR (irrigated control);
2. DT (moderate drought without VG);
3. DT2 (severe drought without VG);
4. VGDT (moderate drought with VG application);
5. VGDT2 (severe drought with VG application).

Experiment 1 included four blocks with four pots per treatment-level combination (total = 40 pots), and Experiment 2 included five blocks with five pots per treatment-level combination (total = 50 pots), for a total of 90 pots across both trials. One plant per pot was grown. The factorial treatment structure and number of replicates in each experiment are outlined in Table 1.

### 2.3. Environmental Data Collection and Monitoring

Microclimatic conditions (temperature and relative humidity) inside the polytunnel were monitored continuously using a Tinytag Gemini Ultra 2 data logger (Gemini Data Loggers, Chichester, UK). The device was mounted at the potato canopy height to best reflect the crop environment. Measurements of air temperature (°C) and relative humidity (%) were recorded automatically at hourly intervals throughout both experiments. These data were subsequently aggregated into weekly means for graphical presentation and interpretation, providing an overview of the prevailing conditions during the study period.

### 2.4. Soil Moisture Measurements, Irrigation Regimes, and Drought Imposition

Soil volumetric water content (VWC) was measured throughout both experiments using a FieldScout TDR100 probe (Spectrum Technologies, Aurora, IL, USA) inserted to a depth of 20 cm. Preliminary water retention characteristics of the John Innes No. 2 compost were determined, indicating a field capacity of approximately 45% VWC and a estimated permanent wilting point (PWP) of about 7.5% VWC [21], equivalent to an available water content (AWC) of approx. 37.5%.

From planting until tuber initiation, all pots received regular irrigation to maintain soil moisture close to 43% VWC (~95% field capacity). In this study, each irrigated plant was supplied with 3 L of water every other day, slightly more than in our earlier experiments [19], where 2 L was used, to prevent inadvertent drought stress in well-watered controls under the warmer seasonal conditions of 2024. Drought was imposed at tuber initiation, 54 days after planting (DAP) in Exp 1 and 38 DAP in Exp 2, which was determined via destructive sampling. The soil-moisture thresholds used to define field capacity, permanent wilting point, available water content, and the irrigation regimes for well-watered and drought treatments are summarised in Table 2.

DT maintained VWC at approximately 18.8% (30% AWC; 70% depletion), producing leaf water potentials of −0.25 to −0.30 MPa. DT2 was imposed by targeting soil VWC below 15% (>80% AWC depletion), with irrigation withheld until soil moisture values approached, but generally remained above the estimated PWP (~7.5% VWC). Under these conditions, leaf water potential fell below −0.40 MPa, indicating severe but sub-PWP water stress.

Soil moisture levels for the DT, DT2, VGDT, and VGDT2 treatments were monitored every 2–3 days using the TDR100 probe to ensure accurate and consistent stress imposition. The timing of drought imposition, VG applications, and physiological measurements in the two experiments is summarised in Table 3.

### 2.5. Foliar Application of Antitranspirant

Vapor Gard (VG, 96% di-1-*p*-menthene, Pinolene^®^, Miller Chemicals and Fertiliser, Hanover, PA, USA), a film-forming product that creates a semi-permeable cuticular barrier to reduce transpirational water loss, was prepared at a concentration of 5 mL L^−1^ in water and applied as a fine foliar mist using a handheld sprayer (Hozelock Exel, Arnas, France) operated at approximately 2 bar pressure. The spray was directed onto the adaxial leaf surfaces until complete coverage was achieved without runoff. Applications were made twice per experiment: the first around 38–42 days after emergence (tuber initiation), and again two weeks later, when soil VWC approached 30% AWC. Untreated controls (IRR, DT, DT2) received no spray.

Spray drift was prevented, and targeted application was achieved by covering neighbouring plants with paper guards and applying sprays during calm morning conditions. The focus on VG was based on prior findings where VG consistently improved plant water status and reduced tuber russeting, whereas abscisic acid (ABA) showed limited effects on physiological disorders [19]. Only Challenger and Russet Burbank were used, as previous experiments identified these varieties as most prone to physiological disorders.

### 2.6. Stomatal Conductance Measurements

Stomatal conductance (gₛ, mmol m^−2^ s^−1^) was measured using an SC-1 Leaf Porometer (Meter Group Inc., Pullman, WA, USA). The instrument was calibrated before each session using the manufacturer’s calibration plate, ensuring an error margin of <5%.

Although VG was applied primarily to the adaxial surface, stomatal conductance was measured on the abaxial surface of the youngest fully expanded leaflet. The abaxial surface has a higher stomatal density and represents the major pathway for water loss in potatoes. Measuring conductance on this surface may underestimate direct VG effects because the abaxial surface received minimal product, but it provides a physiologically meaningful measure of leaf-level drought response. This also reflects field practice, where coverage of the underside is rarely complete.

Three leaflets were measured per pot, and the mean value was used for analysis. Readings were collected between 10:00 a.m. and 1:00 p.m., the period of highest evaporative demand. Although stomata may partially close by midday under drought, this window provides the most informative assessment of drought regulation. Measurements were taken once prior to drought initiation and then every 2–3 days during the stress period until harvest.

### 2.7. The Relative Water Content (RWC) Determination

RWC was determined using the standard fresh–turgid–dry mass method. Fully expanded upper-canopy leaflets were sampled and immediately placed in airtight bags inside a cooled container.

- Fresh weight (FW): measured within 1 h
- Turgid weight (TW): after 24 h immersion at 4 °C
- Dry weight (DW): after 48 h at 80 °C

RWC (%) = [(FW − DW)/(TW − DW)] × 100

Measurements were taken at 77 DAP (Exp 1) and 56 DAP (Exp 2) during early bulking.

### 2.8. Yield Assessment and Evaluation of Tuber Physiological Disorders

At physiological maturity, all tubers were hand-harvested, washed, and air-dried for 24 h before their total fresh weight and the number of tubers per pot were recorded using a high-precision digital balance (KERN FKB 16K0.1, KERN & Sohn GmbH, Balingen, BW, Germany). Mean tuber weight was calculated per pot.

For tuber quality evaluation, 6–8 randomly selected tubers per plant were assessed for the presence or absence of three physiological disorders: russeting, internal brown spot/necrosis, and jelly end rot (JER).

- Russeting was recorded when >5% of the skin surface showed roughness or cracking.
- Internal disorders were assessed by longitudinal cutting and visual inspection for clearly visible brown or necrotic lesions [22,23].

### 2.9. Statistical Analysis

Data processing employed Genstat 23rd edition (VSN International, Hemel Hempstead, UK; https://vsni.co.uk/software/genstat/ (accessed on 10 January 2024)) and R software version 4.4.1 (R Core Team, Vienna, Austria; https://www.r-project.org/ (accessed on 28 June 2025)) following protocols adapted from [19]. Treatment effects were evaluated via two-way ANOVA with predefined orthogonal contrasts to test specific hypotheses. The standard treatment order used for all analyses was IRR, DT, DT2, VGDT and VGDT2, representing increasing drought intensity and subsequent VG application. The four orthogonal contrasts were defined as follows:

1. IRR vs. (DT + DT2 + VGDT + VGDT2): overall effect of drought compared with full irrigation.
2. (DT + DT2) vs. (VGDT + VGDT2): overall effect of VG under drought conditions.
3. DT vs. DT2: effect of increasing drought severity without VG.
4. VGDT vs. VGDT2: effect of increasing drought severity under VG application.

Repeated-measures ANOVA was used for the soil moisture content and stomatal conductance data, as these variables were measured over time. Model residuals were checked for normality using histograms and Q–Q plots, and homoscedasticity was verified by examining the residuals versus the fitted values. Where relevant, group estimates are reported as means with accompanying standard errors of difference (SED). To assess the treatment effects on the incidence of physiological disorders, Fisher’s exact test was applied separately for each potato variety, using the same contrast structure. This approach was chosen due to the small, unbalanced sample sizes, thereby ensuring robust inference.

## 3. Results

### 3.1. Observed Environmental Conditions in the Polytunnel

**Figure 1:**
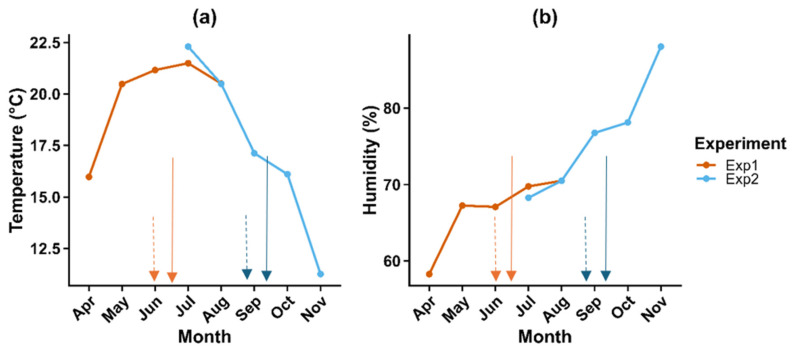
(**a**) Daily mean air temperature (°C) and (**b**) daily mean relative humidity (%) for Experiment 1 (orange line, April–August 2024) and Experiment 2 (blue line, July–November 2024) recorded during their respective potato growing seasons. Solid arrows indicate tuber initiation, when drought treatments were imposed, and dashed arrows indicate Vapor Gard application, which occurred 2 weeks after tuber initiation in each experiment (orange arrows: Experiment 1; blue arrows: Experiment 2). Tick marks on the x-axis denote the start of each month. Experiment 1 commenced on 12 April 2024, and Experiment 2 on 24 July 2024.

Seasonal profiles for air temperature and relative humidity (RH) differed markedly between Exp 1 and Exp 2 (Figure 1 and Table 4).

#### 3.1.1. Experiment 1

Daily mean air temperature ranged from 10.8 to 29.7 °C (seasonal mean 20.2 °C). Minimum temperatures often fell below 10 °C during April and early May, while maximum values exceeded 35 °C during several episodes in late June and early July. Short-term temperature variability was high, with increases of >10 °C between consecutive days recorded in late June. RH was highly variable, ranging from 38.3% to 96.2% (mean 67.3%), with the lowest values (<35%) coinciding with hot, dry days in late June (Figure 1; Table 4).

#### 3.1.2. Experiment 2

Mean daily air temperature was slightly lower and more stable overall, ranging from 9.4 to 25.5 °C (seasonal mean 17.4 °C). Maximum temperatures exceeded 25–27 °C during late July and August, but overall fluctuations were less pronounced than in Exp 1. RH was consistently higher and more stable, ranging from 52.6% to 99.9% (mean 76.2%). Values above 70% predominated through much of August and September, with maximum RH reaching or exceeding 100% overnight, reflecting reduced evaporation and frequent dew formation as day length declined (Figure 1; Table 4).

### 3.2. Volumetric Water Content (VWC)

VWC varied significantly across treatments in both experiments (*p* < 0.001; Table 5), with droughted pots consistently lower than irrigated controls. Significant effects of time and treatment-by-time interaction (*p* < 0.001 for both experiments) were detected. A significant variety effect was detected in Exp 2 (*p* = 0.013), whereas the effect was not significant in Exp 1 (*p* > 0.05), and almost no interactions with variety occurred in both experiments.

#### 3.2.1. Experiment 1

**Figure 2:**
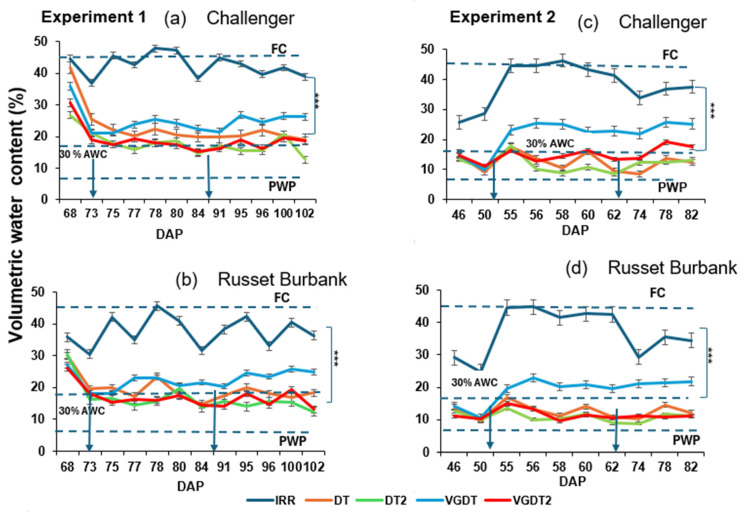
Changes in volumetric water content (VWC, %) over time in (**a**,**c**) Challenger and (**b**,**d**) Russet Burbank potatoes under five treatments: irrigated control (IRR), moderate drought without Vapor Gard (DT), severe drought without Vapor Gard (DT2), moderate drought with Vapor Gard (VGDT), and severe drought with Vapor Gard (VGDT2) during tuber initiation and after spraying antitranspirants. Arrows represent the days of spraying VG. The target VWC of water stress treatments is 30% AWC for moderate drought treatments (DT and VGDT); severe drought treatments (DT2, VGDT2) were maintained at or near the permanent wilting point (PWP) (often closer to 0–10% AWC). Dashed horizontal lines indicate field capacity (FC), the 30% AWC threshold and the estimated PWP. Data are means of replicates (*n* = 4 for Experiment 1; *n* = 5 for Experiment 2). Error degrees of freedom: df = 27 (Experiment 1), df = 36 (Experiment 2). Error bars represent the Standard Error of Difference (SED). FC is Field Capacity, and PWP is Permanent Wilting Point, where IRR = Irrigated, DT = Drought, DT2 = Severe Drought and VG = Vapor Gard. Asterisks (***) represent significant differences in IRR compared to DT and ATs according to Tukey’s test (*p* = 0.05). Drought stress was imposed at tuber initiation (54 DAP in Exp 1 and 38 DAP in Exp 2).

VWC in IRR pots remained close to field capacity (40–45% VWC). Upon drought imposition, VWC declined rapidly: DT caused a 30% reduction, and DT2 approximately 40% compared with IRR. After the application of the film-forming antitranspirant, under moderate drought, VGDT increased VWC by 5–10% compared with untreated DT, narrowing the gap with IRR pots. In contrast, under severe drought treatment, VGDT2 averages approximately 28% VWC, similar to DT2 (Figure 2a,b).

#### 3.2.2. Experiment 2

VWC in IRR pots was lower than in Exp 1, averaging 38–40%, due to the higher ambient temperatures and evapotranspirative demand. Moderate DT reduced VWC by approximately 50% compared to IRR, and severe drought treatment (DT2) by over 55%. VG application under moderate drought (VGDT) slightly increased soil VWC (<5% higher than DT), but this difference was not sustained as stress progressed. Under severe drought, VWC in VGDT2 remained close to DT2 and declined to near the estimated permanent wilting threshold (~7.5%), while generally remaining above this level, indicating severe but sub-PWP soil water limitation rather than sustained permanent wilting (Figure 2c,d).

### 3.3. Stomatal Conductance (SC)

SC varied significantly across treatments in both experiments (*p* < 0.001; Table 5), with drought stress resulting in large reductions compared with irrigated controls. Significant effects of time (*p* < 0.001) and treatment × time interactions (*p* ≤ 0.02) were also detected. In Exp 1, variety and the variety × treatment interaction were significant for SC (*p* < 0.001 and *p* = 0.036, respectively), whereas in Exp 2, neither variety nor its interactions were significant (*p* > 0.05).

#### 3.3.1. Experiment 1

**Figure 3:**
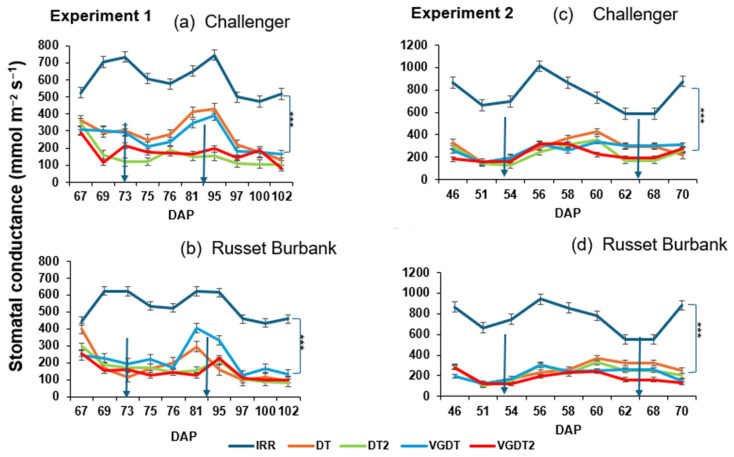
The stomatal conductance for Exp 1 and 2 for (**a**,**c**) Challenger and (**b**,**d**) Russet Burbank pots under irrigated (IRR) and water-stressed (DT, DT2, VGDT and VGDT2) conditions before and after spraying antitranspirants. Arrows represent the day of spraying Vapor Gard antitranspirant. Data are means of replicates (*n* = 4 and 5 for Exp 1 and 2, respectively). Error bars represent the Standard Error of Difference (SED). DF = 27 (Exp 1); df = 36 (Exp 2). FC is the Field Capacity, and PWP is the Permanent Wilting Point. Where IRR = Irrigated, DT = Drought, DT2 = severe stress and VG = Vapor Gard, Asterisks (***) represent significant differences between IRR and drought/VG treatments.

The mean SC under irrigation was 568 mmol m^−2^ s^−1^. Stomatal conductance was reduced by approximately 59% with DT and by approximately 72% with DT2, relative to IRR (Figure 3a,b). The application of VG under moderate DT resulted in a small increase in SC (~4% above DT), indicating a small treatment effect. Under severe drought, VGDT2, SC remained 72% below IRR, statistically similar to DT2. Contrasts revealed significant effects across all comparisons, including IRR vs. DTs, DT vs. DT2, DTs vs. VGDTs, and VGDT vs. VGDT2 (all *p* < 0.001), confirming the strong influence of both drought and VG application on SC.

#### 3.3.2. Experiment 2

The mean SC under irrigated plants was 762 mmol m^−2^ s^−1^. Drought stress again sharply reduced SC: Stomatal conductance was reduced by approximately 64% in DT plants and approximately 69% in DT2 plants, relative to IRR. VG application under moderate drought caused a small decrease in SC (~11% below DT). Under severe drought, VGDT2 decreased SC by approximately 12% relative to DT2. However, both remained >70% below IRR, indicating that drought imposed substantial stomatal limitation regardless of VG treatment (Figure 3c,d). Contrasts again showed significant differences between IRR vs. DTs, DTs vs. VGDTs, and VGDT vs. VGDT2 (all *p* < 0.001), but not between DT and DT2 (*p* = 0.564).

### 3.4. Relative Water Content (RWC)

**Figure 4:**
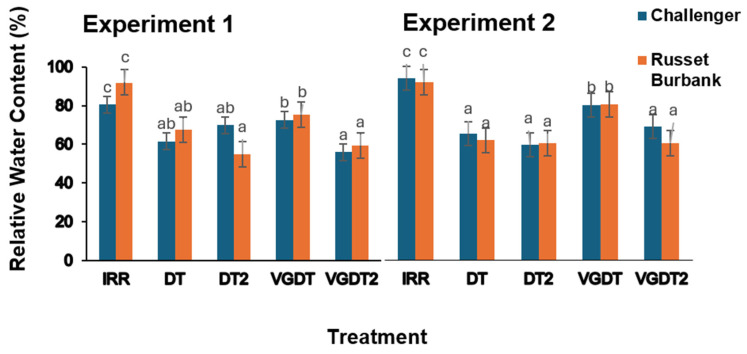
Relative water content (RWC) of two potato varieties, Challenger and Russet Burbank, under different irrigation and antitranspirant treatments across Experiments 1 and 2. Treatments included irrigated control (IRR), drought (DT), severe drought (DT2), and Vapor Gard antitranspirant application (VGDT, VGDT2). Bars represent mean RWC (±standard error) for each treatment and variety (blue = Challenger; orange = Russet Burbank), with *n* = 4 replicates in Experiment 1 and *n* = 5 in Experiment 2. Different letters above bars indicate statistically significant differences (*p* < 0.05) within each experiment and variety.

RWC varied significantly across treatments in both experiments (*p* < 0.001, Table 5, Figure 4). Variety and variety × treatment interactions were not significant (*p* > 0.05), suggesting a consistent response pattern across varieties.

#### 3.4.1. Experiment 1

Moderate DT reduced RWC by 33% relative to the irrigated control, while DT2 reduced RWC by 35%. VGDT increased RWC by 23% compared with DT. However, under severe drought, VGDT2 maintained RWC values similar to DT2, with both treatments averaging approximately 59% of IRR. Contrast analysis confirmed significant differences between IRR and DTs (*p* = 0.012) and between DT and DT2 (*p* < 0.001), as well as between VGDT and VGDT2 (*p* < 0.002), whereas the contrast between DTs and VGDTs was marginally significant (*p* = 0.053).

#### 3.4.2. Experiment 2

RWC was reduced by 32% under moderate DT and by 36% under DT2 compared with IRR. VG application under moderate drought significantly improved RWC, increasing it by approximately 27% relative to DT (*p* < 0.001); however, RWC values remained 14% lower than IRR. Under severe drought, RWC in VGDT2 did not differ significantly from DT2, despite a small numerical increase of approximately 8%. Contrast analysis revealed significant differences between IRR and DTs, between DTs and VGDT, and between VGDT and VGDT2 (*p* < 0.001).

### 3.5. Yield

Tuber yield varied significantly across treatments in both experiments (*p* < 0.001, Table 6). Variety effects and treatment × variety interactions were not significant (*p* > 0.05), indicating similar treatment responses in both varieties.

#### 3.5.1. Experiment 1

Irrigated plants produced the highest yield (1579 g/plant). DT and DT2 reduced yield to 653 and 611 g/plant, respectively (59–61% lower than IRR). VGDT increased yield to 1090 g/plant (67% higher than DT; contrast DTs vs. VGDTs, *p* = 0.010) and narrowed the gap with irrigated plants. Under severe drought, VGDT2 yielded 740 g/plant (21% higher than DT2) but still significantly lower than VGDT (VGDT vs. VGDT2 *p* < 0.001) (Table 6).

#### 3.5.2. Experiment 2

Irrigated plants again had the highest yield (1559 g/plant). DT and DT2 reduced yield to 1100 g/plant and 985 g/plant, respectively (29–37% reductions relative to IRR). VG application under moderate DT increased yield to 1504 g/plant (37% increase relative to DT), though the improvement was borderline statistically significant (*p* = 0.06). Under severe drought, VGDT2 yielded 1117 g/plant, which was significantly lower than VGDT (VGDT vs. VGDT2 *p* < 0.001).

### 3.6. Potato Physiological Disorders

The incidence of tuber physiological disorders (russeting, internal brown spot/necrosis, and jelly end rot) varied significantly across treatments in both experiments (*p* < 0.001; Table 7, Table 8 and Table 9). Because disorder patterns differed between varieties, the results are described separately for each variety.

#### 3.6.1. Experiment 1: Challenger

Under IRR, 88% of tubers were disorder-free, with russeting at 13% and internal brown spot/necrosis at 4% (Table 7). DT reduced the proportion of disorder-free tubers to 17% and increased russeting to 71%. VGDT increased the proportion of disorder-free tubers to 67% and reduced russeting to 33%, with no necrosis observed. DT2 resulted in 42% disorder-free tubers and 58% russeting. VGDT2 resulted in 62% disorder-free tubers, 29% russeting, and 8% necrosis. VGDT and VGDT2 did not differ significantly for any disorder (all *p* > 0.25).

#### 3.6.2. Experiment 1: Russet Burbank

Under IRR, 75% of tubers were disorder-free; russeting was absent, while necrosis and jelly end rot were 17% and 8%, respectively (Table 7). DT reduced the number of disorder-free tubers to 33% and increased necrosis to 46%. DT2 further increased necrosis to 50% and russeting to 25% (Table 7). VGDT increased disorder-free tubers to 67% and reduced necrosis to 17% (DTs vs. VGDTs: necrosis *p* < 0.001; russeting *p* = 0.030; Table 8). VGDT2 resulted in 58% disorder-free tubers with necrosis at 17% and russeting at 13%; VGDT vs. VGDT2 differences were not significant (all *p* > 0.25)

#### 3.6.3. Experiment 2: Challenger

Under IRR, 90% of tubers were disorder-free, and 10% had russeting (Table 9). DT reduced the proportion of disorder-free tubers to 32% and increased russeting to 62%, while DT2 reduced the proportion of disorder-free tubers to 8% and increased russeting to 82%. VGDT increased disorder-free tubers to 85% and reduced russeting to 12% (DTs vs. VGDTs: *p* < 0.001 for russeting and disorder-free). VGDT2 resulted in 50% disorder-free tubers and 45% russeting; russeting remained higher than in VGDT (VGDT vs. VGDT2: *p* < 0.001).

#### 3.6.4. Experiment 2: Russet Burbank

Under IRR, 95% of tubers were disorder-free, with necrosis at 5% and no russeting or jelly end rot. DT reduced disorder-free tubers to 28% and increased jelly end rot to 40% (Table 9). VGDT increased disorder-free tubers to 78% and reduced jelly end rot to 2% (DTs vs. VGDTs: jelly end rot *p* < 0.001; disorder-free *p* = 0.001; russeting *p* < 0.001). Under severe drought, VGDT2 resulted in 70% disorder-free tubers with necrosis at 30% and no jelly end rot; VGDT vs. VGDT2 differences were not significant overall, although necrosis was borderline (*p* = 0.051).

## 4. Discussion

Drought stress is a major abiotic constraint on potato production, and yield losses under severe water limitation can be substantial [24,25]. In the present study, drought reduced plant water status, stomatal conductance, and tuber yield in both experiments, and increased the incidence of tuber physiological disorders. These responses confirm that soil water deficit during tuber initiation and bulking compromises both productivity and tuber quality in potato.

This study evaluated whether a film-forming antitranspirant (Vapor Gard, VG) could reduce drought-induced effects on plant water status, yield, and tuber physiological disorders in two potato varieties under moderate and severe soil drought. VG offered measurable protection primarily under moderate drought, increasing relative water content, maintaining yields closer to those with irrigation, and decreasing the occurrence of certain physiological disorders. However, its benefits lessened under severe drought, supporting the idea that VG effectiveness depends on both the level of stress and environmental conditions.

The following sections explore these findings in detail, evaluating the role of VG in maintaining water relations, modulating stomatal behaviour, sustaining yield, and reducing physiological disorders, while considering varietal differences and assessing the associated null hypotheses.

### 4.1. Influence of Drought Severity and Vapor Gard on Relative Water Content

RWC is a widely accepted indicator of leaf hydration and drought severity. In both experiments, drought significantly reduced RWC compared with irrigation, confirming that soil water deficit lowered leaf hydration and refuting the null hypothesis that drought does not affect leaf water status. Moderate stress (DT) caused 25–30% reductions, while severe drought (DT2) exacerbated the loss, consistent with the 25–35% decline reported for potatoes under drought [24]. Accordingly, RWC is interpreted here as a relative indicator of plant hydration under imposed soil water deficits, rather than a direct measure of plant water potential or varietal hydraulic capacity.

The effect of VG varied between experiments. In Exp 1, VG application under moderate drought produced a modest but significant increase in RWC (~14% greater than DT), indicating partial improvement in leaf hydration under high evaporative demand. However, under severe drought, VG offered no benefit (VGDT2 = DT2), confirming its limited effectiveness when soil moisture drops below a critical threshold. Conversely, in Exp 2, VG produced a stronger response, significantly increasing RWC (~27% greater than DT) under moderate drought, rejecting the null hypothesis and showing a protective effect when environmental conditions were milder and humidity was higher. Under severe drought, VGDT2 again did not differ from DT2, reinforcing that VG’s benefits are confined to moderate stress levels.

These contrasting outcomes reflect mainly environmental conditions. Exp 1 experienced lower relative humidity and high vapour pressure deficit (VPD), conditions known to increase atmospheric evaporative demand and impose hydraulic strain on the plant, leading to faster declines in leaf water status [25]. These same conditions also likely accelerate drying of the VG film. Rapid desiccation is known to promote stress accumulation and microcrack formation in thin polymer coatings [26], thereby reducing VG persistence. In contrast, Exp 2 occurred under more humid, thermally stable conditions that enhanced film adhesion and durability, thereby improving water retention [27]. Moderate and stable VPD has also been shown to sustain higher stomatal conductance and photosynthetic activity compared with rapid VPD fluctuations [28], supporting the interpretation that steady humidity favours canopy water balance and enhances VG efficacy.

Elevated leaf-to-air VPD can also induce substantial water stress even when soil moisture remains adequate, as transpirational demand may exceed root uptake and xylem transport capacity, leading to hydraulic imbalance and leaf dehydration [29]. Furthermore, because VG was primarily applied to the adaxial surface, where stomatal density is lower, the abaxial stomata likely remain uncovered, leading to continued water loss through transpiration. Based on prior comparative measurements in potato (unpublished), and supported by published literature, the abaxial leaf surface is the dominant route for water loss due to much higher stomatal density [27,29]. These factors collectively explain the weaker VG performance under the hotter, high-VPD conditions of Exp 1. These interpretations are based on observed plant responses and environmental context, rather than direct measurements of film persistence, stomatal mechanics, or hydraulic conductance.

Varietal differences in RWC were not significant, supporting the null hypothesis that varieties did not differ in water status. This aligns with previous studies suggesting that genotypic variation in potato RWC is minor compared with treatment effects [30,31]. The consistent responses suggest that VG’s effect is mainly a physical film-forming function rather than mediated by any genotype-specific physiological mechanisms of drought tolerance.

Overall, these findings indicate that VG’s effectiveness in maintaining leaf hydration depends strongly on the environmental context. During moderate drought, sustaining higher RWC can delay cellular and metabolic stress, helping to preserve leaf function and yield potential. However, in severe drought conditions, when both soil moisture and atmospheric humidity are critically low, the film barrier alone may be insufficient. Growers might apply VG during anticipated moderate stresses or integrate it with soil moisture conservation practices during severe drought, rather than as a substitute for irrigation under severe drought.

### 4.2. Stomatal Conductance (SC) Responses to Drought and Vapor Gard Application

SC is a key physiological trait regulating transpiration and plant water relations under drought stress. Our study in both experiments demonstrates that drought significantly reduced SC compared with irrigation, refuting the null hypothesis that drought does not affect SC. The decline in SC reflects a typical drought avoidance response, where stomata close to conserve water and maintain leaf water potential. This is consistent with established potato drought physiology [19,24,27,29].

In Exp 1, SC decreased by 59% under DT and 72% under DT2 relative to IRR, confirming that increasing drought severity further restricts stomatal aperture. The contrast analysis indicated a statistically significant effect of VG application under moderate drought (DTs vs. VGDTs), with VGDT showing a small but significant increase in SC (approximately 4% higher than DT). This refutes the null hypothesis that VG does not affect SC under moderate stress, suggesting a weak or transient physiological influence. However, under severe drought (DT2 vs. VGDT2), SC did not decrease further with VG and was statistically indistinguishable from DT2, supporting the null hypothesis that VG has no additional effect once stomatal closure is maximal.

This pattern reflects a methodological constraint: VG was applied mainly to the adaxial surface, while SC was measured exclusively on the abaxial surface, which is the dominant route of water loss in potato [27,29]. This literature indicates that film-forming antitranspirants such as VG provide the most significant reduction in SC when adequately covering the abaxial surface. If coverage is incomplete or the film is not stable, especially under high VPD, observed effects are minor, as seen here. Accordingly, SC is interpreted here primarily as an indicator of drought severity rather than as direct evidence of VG–mediated stomatal regulation.

In Exp 2, SC showed a similar pattern, decreasing by 64% under DT and 69% under DT2, compared with IRR, refuting the null hypothesis that drought does not affect SC. The contrast between DT and VGDT was statistically significant for SC, with a small 11% decrease (VGDT vs. DT). However, the difference was minor and of questionable biological relevance, providing only weak evidence against the null hypothesis that VG alters SC under moderate drought. This transient effect is consistent with prior results for antitranspirant films under fluctuating growing conditions. Under severe drought, VGDT2 and DT2 were again statistically similar, supporting the null hypothesis that VG does not impact SC after full endogenous stomatal closure is achieved [27,29].

The observed VG effect on SC differed between experiments (a slight increase in Exp 1, a slight decrease in Exp 2); both effects were small and of limited biological relevance. These minor changes likely reflect a combination of environmental context and methodological limitations, indicating that VG, as applied here, had little practical impact on abaxial stomatal conductance in droughted potato [19,27]. As film persistence and surface coverage are critical for efficacy, these findings underscore the need for methodologies that align application and measurement surfaces in future studies.

### 4.3. Yield Responses to Drought Severity and Vapor Gard Application

Potato yield is highly sensitive to water deficits, particularly during tuber initiation and bulking, when assimilate supply and partitioning determine the final number and size of tubers. Consistent with previous research showing yield reductions of 20–60% under moderate to severe drought [30,31], both experiments demonstrated strong adverse effects of drought on tuber yield, refuting the null hypothesis that drought does not affect yield. In Exp 1, yield declined by more than 60% under severe drought (DT2) compared with irrigation, while in Exp 2, the losses were smaller, but still substantial. These differences likely reflect seasonal environmental variation: Exp 1 was characterised by higher evaporative demand and lower RH, exacerbating water stress, whereas Exp 2 experienced more stable, humid conditions that partially buffered drought effects.

The VG application provided measurable mitigation under moderate drought conditions in both experiments. VGDT significantly increased yield by around two-thirds over DT in Exp 1 and by about one-third over DT in Exp 2, refuting the null hypothesis that VG does not affect yield during drought. The improvement was most notable in Exp 2, where VGDT restored yields to nearly irrigated levels, while in Exp 1, the benefit was less substantial. These findings support the idea that VG can delay or buffer the effects of drought when soil water is limited but not entirely exhausted, aligning with results in rapeseed and wheat, where film antitranspirants improved yield under moderate stress but not in severe conditions [32,33]. Under severe drought (VGDT2), yields remained similar to untreated DT2, supporting the null hypothesis that VG cannot replace irrigation at critical water deficits; therefore, yield could not be sustained once soil moisture falls below the physiological compensation threshold. This plateau in effectiveness likely reflects both physical and physiological limits, as water potential declines: stomatal closure, photosynthetic inhibition, and assimilate restriction collectively constrain yield formation [24,34]. Beyond this point, a surface film alone cannot offset the internal hydraulic and metabolic constraints on tuber development. In the absence of measurements of photosynthetic rate, chlorophyll fluorescence, and biomass accumulation, yield responses are interpreted here as integrated outcomes of drought stress and mitigation rather than as evidence of specific carbon assimilation mechanisms.

Varietal responses were consistent across experiments, with Challenger generally producing higher yields than Russet Burbank, but no significant treatment × variety interactions were detected. This indicates that both cultivars benefited similarly from VG application, supporting the null hypothesis of no genotype-specific differences in yield response under these conditions. Nonetheless, the absolute yield reductions differed: Russet Burbank experienced greater proportional losses under drought, consistent with its reputation for weaker stress tolerance [30]. These differences underline the importance of integrating varietal resilience with antitranspirant-based mitigation strategies.

### 4.4. Impact of Vapor Gard on Physiological Disorders in Potato Tubers

Physiological disorders such as russeting, necrosis, and jelly end rot represent significant constraints to potato quality, reducing marketability and storability. In our study, across both experiments, drought markedly increased disorder incidence, refuting the null hypothesis that drought does not affect tuber quality.

#### 4.4.1. Russeting

Russeting emerged as the most drought-sensitive disorder, particularly in Challenger, aligning directly with the central research question of this study. In both experiments, drought substantially increased the incidence of russeting and reduced the proportion of disorder-free tubers. VG application under moderate drought significantly reduced russeting, with VGDT plants in both experiments showing markedly lower russeting percentages than untreated DT plants. These findings refute the null hypothesis that VG does not alter disorder incidence and demonstrate that VG can act as a protective tool against russeting under moderate stress.

#### 4.4.2. Internal Brown Spot/Necrosis

Internal necrosis also increased under drought, especially in Russet Burbank. VG application reduced necrosis under moderate drought in both varieties, though the reductions were less pronounced than those for russeting. Under severe drought, necrosis remained elevated regardless of VG application, supporting the conclusion that VG’s protective effects weaken as stress intensifies.

#### 4.4.3. Jelly End Rot

Jelly end rot occurred only in Russet Burbank, where drought significantly increased its incidence (up to 40% under DT in Exp 2). VG application substantially reduced jelly end rot under moderate drought, dropping from 40% to 2.5% in Exp 2; however, its benefit again diminished under severe drought. This confirms that VG is most effective when drought is moderate rather than extreme.

Varietal predispositions were evident: Challenger was more susceptible to russeting, and Russet Burbank was more vulnerable to necrosis and jelly end rot. Although not always statistically significant, these differences suggest genotype-specific vulnerabilities, partially refuting the null hypothesis that varieties do not differ in the disorders.

These results align with earlier reports showing that drought stress increases russeting and other tuber disorders specifically in potato by disrupting periderm development and phellem cohesion, characterised by skin patches with withered skin cells attached to the new skin underneath [7,8,35]. Similar mechanisms have been described for internal potato disorders, such as jelly end rot, which are linked to sugar accumulation under stress [36,37]. Importantly, our findings are consistent with our previous research on potatoes, where VG reduced russeting under drought [19], suggesting that VG-associated improvements in whole-plant water status coincide with reduced russeting during tuber expansion during moderate drought. Direct measurements of tuber water content were not included in this study; therefore, the interpretation is based on whole-plant physiological status rather than direct tuber-level mechanisms.

The consistent reduction of russeting by VG under moderate drought highlights its potential as a targeted management tool in environments where water stress is common but not extreme. For growers, this suggests that VG can improve marketable yield by maintaining smoother skins in susceptible varieties, such as Challenger. However, the limited efficacy under severe drought emphasises that VG should be considered a complementary strategy rather than a substitute for irrigation or other water-conservation practices.

### 4.5. Indicative Economic Implications of Vapor Gard Under Moderate Drought

To illustrate the potential economic relevance of Vapor Gard application under moderate drought, an indicative cost–benefit scenario was developed using observed yield responses and representative UK market values. Mean yield gains associated with VG application under moderate drought (VGDT vs. DT) were 437 and 404 g/plant in Exps 1 and 2, respectively (Table 6). Because the experiments were conducted at low planting density (approximately one plant/m^2^, equivalent to ~10,000 plants/ha), yield responses were extrapolated using this experimental density rather than a typical commercial field density. On this basis, the observed yield gains correspond to indicative increases of approximately 4.4 and 4.0 t/ha in Exps 1 and 2, respectively.

Farm-gate potato prices in the UK vary seasonally and by market class; industry reports indicate that typical ex-farm prices commonly fall within a broad range of approximately £250–£350/t across seasons and production systems [38]. Using a conservative mid-range value (£270/t), the corresponding gross revenue associated with these yield gains would be approximately £1080–£1180/ha. Application costs were estimated using published values for di-1-*p*-menthene application in arable crops, including product cost and a standard UK spray pass, giving a total cost of approximately £26.54/ha [20]. On this basis, the break-even yield increase required to justify VG application is approximately 0.10 t/ha (100 kg/ha). Yield responses observed under simplified experimental conditions may exceed those achievable in well-managed field crops; therefore, these values should be regarded as illustrative rather than predictive [39]. Nevertheless, the analysis indicates that even substantially smaller yield benefits than those observed here could economically justify VG application under moderate drought, consistent with previous evaluations of film-forming antitranspirants in drought-stressed wheat [20].

### 4.6. Study Limitations and Implications for Application

Several limitations constrain the generalisation of the findings and should be considered when interpreting the results. Microclimate drivers of VG performance, including VPD, photosynthetically active radiation, and wind speed, were not quantified, limiting the mechanistic explanation of between-experiment differences. Film presence, coverage uniformity, and persistence were not directly measured; therefore, VG effects cannot be attributed specifically to film dynamics over time. Stomatal conductance was measured on the abaxial leaf surface, whereas VG was applied primarily to the adaxial surface, limiting mechanistic inference regarding gas-exchange responses. In addition, tuber physiological disorders were assessed as incidence (presence/absence) rather than severity, which may reduce sensitivity to treatment effects. Direct measurements of tuber water status and periderm development (e.g., tuber water content or anatomical markers) were not undertaken; consequently, reductions in physiological disorders are interpreted as practical outcomes associated with improved whole-plant water status rather than evidence of a specific mechanistic pathway. Finally, pot culture under polytunnel conditions limits extrapolation to field systems with natural soils and variable weather.

Despite these constraints, a consistent pattern emerged in the two experiments reported here, and this pattern is consistent with findings from our previously published experiments [19], indicating that VG provides the greatest benefit under moderate soil water deficit, improving plant water status, maintaining yields closer to irrigated levels, and reducing key quality losses, particularly russeting. Future research should evaluate responsive application strategies triggered by soil or plant water status, quantify film persistence under contrasting environments, and validate VG performance under field conditions to support practical adoption.

## 5. Conclusions

This study demonstrates both the potential and the limitations of VG as a drought-mitigation tool in potatoes. VG improved relative water content and consistently reduced russeting under moderate soil water deficit; however, its benefits diminished under severe drought, highlighting that VG’s effectiveness is strongly dependent on drought intensity and environmental conditions. Varietal predispositions affected the incidence of disorder but did not alter VG’s overall protective role. These findings indicate that VG can serve as a complementary management strategy to reduce drought-related quality losses, particularly russeting, under moderate drought conditions, but cannot substitute for irrigation under severe water limitations. Further research is required to optimise application timing, assess film persistence, and validate performance under field conditions.

## Figures and Tables

**Table 1:** Orthogonal contrast structure used to test treatment effects in Experiments 1 and 2. The four planned contrasts evaluated the overall impact of drought, the effect of Vapor Gard (VG) under drought, and drought severity with and without VG. IRR = irrigated control; DT = moderate drought; DT2 = severe drought; VGDT = moderate drought + VG; VGDT2 = severe drought + VG.

Contrast No.	Comparison	Description
1	IRR vs. (DT + DT2 + VGDT + VGDT2)	Overall effect of drought
2	(DT + DT2) vs. (VGDT + VGDT2)	Effect of VG under drought
3	DT vs. DT2	Effect of drought severity without VG
4	VGDT vs. VGDT2	Effect of drought severity under VG

**Table 2:** Soil moisture measurement thresholds for John Innes No. 2 compost, showing field capacity (FC), permanent wilting point (PWP), available water content (AWC), and irrigation regimes for well-watered and drought treatments. All percentages are volumetric water content (VWC) by soil volume.

Parameter	% of Soil Measurement	Notes
Field Capacity (FC)	45% VWC	-
Permanent Wilting Point	7.5% VWC	–
AWC	37.50%	FC − PWP
Well-watered	42.75% VWC (95% FC)	IRR
DT threshold	18.8% VWC (30% AWC)	Moderate drought
DT2 threshold	~10–15% VWC (>80% AWC depletion)	Severe drought

**Table 3:** Overview of irrigation protocols, antitranspirant treatments, and key physiological measurements conducted in Experiments 1 and 2.

Experiment 1	Experiment 2					
Week	Week	Stage	DAP Drought Initiation	% of AWC	AT	Assessments
1–4	1–2	Establishment		100% of	-	-
5–8	3–5	Stolon initiation		100%	-	-
8–12	5–9	Tuber initiation	54 (Exp 1) 38 (Exp 2)	Dry-down	-	Porometer
10 (June 25)	7 (September 13)	Tuber initiation		30%	Spray 1	Porometer
11	8	Tuber initiation		30%		Porometer/RWC
12	9	Tuber initiation		30%	Spray 2	Porometer
13–17	10–13	Tuber filling		30%	-	-
18–21	14–17	Maturity		0%	-	-
21	17	Maturity		0%	-	Harvest

**Table 4:** Seasonal summary of daily air temperature (°C) and relative humidity (%) in polytunnel conditions during Experiment 1 (April–August 2024) and Experiment 2 (July–October 2024). Values represent minimum, maximum, and seasonal means calculated from daily observations.

Experiment	Condition	Min	Max	Mean
Exp 1	Temperature	10.8	29.7	20.2
Exp 1	RH	38.3	96.2	67.3
Exp 2	Temperature	9.4	25.5	17.4
Exp 2	RH	52.6	99.9	76.2

**Table 5:** Probability values from ANOVA for volumetric water content (VWC) and stomatal conductance (gs), and Relative water content (RWC) as affected by irrigation (IRR) and Vapor Gard antitranspirant (AT) in two experiments. Bold numbers indicate significant differences at *p* < 0.05.

Experiment	Factors	d.f.			*p* Values		
		VWC	gs	RWC	VWC	gs	RWC
Exp 1	Treatment	4	4	4	**<0.001**	**<0.001**	**<0.001**
	Variety	1	1	1	0.073	**<0.001**	0.874
	Time	11	9	-	**<0.001**	**<0.001**	
	Time × Treatment	44	36	-	**<0.001**	**0.012**	-
	Time × Variety	11	9	-	0.547	0.985	-
	Time × Treatment × Variety	44	36	-	0.979	0.998	-
	CV (%)	4.5	13	4.5			
	Contrast *p* values						
	IRR vs. DTs	1	1	1	**<0.004**	**<0.001**	**0.012**
	DT vs. DT2	1	1	1	**<0.001**	**<0.001**	**<0.001**
	DTs vs. VGDTs	1	1	1	**<0.001**	**<0.001**	0.053
	VGDT vs. VGDT2	1	1	1	**<0.001**	**<0.001**	**0.002**
	Interaction contrast *p* values						
	Variety × Treatment	4	4	4	0.707	**0.036**	0.664
	Variety × IRR vs. DTs	1	1	1	0.858	**0.013**	0.755
	Variety × DTs vs. VGDTs	1	1	1	0.393	0.227	0.377
	Variety × DT vs. DT2	1	1	1	0.681	0.239	0.234
	Variety × VGDT vs. VGDT2	1	1	1	0.283	0.244	0.958
	Time × Treatment	44	36	-	**<0.001**	**0.02**	-
	Time × IRR vs. DTs	11	9	-	**0.004**	0.379	-
	Time × DT vs. DT2	11	9	-	**0.001**	0.104	-
	Time × DTs vs. VGDTs	11	9	-	**0.023**	0.477	-
	Time × VGDT vs. VGDT2	11	9	-	**<0.001**	**0.005**	-
	Time × Variety	11	9	-	0.547	0.985	-
	Time × Variety × Treatment	44	36	-	0.979	0.998	-
	Time × Variety × IRR vs. DTs	11	9	-	0.949	0.464	-
	Time × Variety × DT vs. DT2	11	9	-	0.47	0.98	-
	Time × Variety × DTs vs. VGDTs	11	9	-	0.872	0.992	-
	Time × Variety × VGDT vs. VGDT2	11	9	-	0.917	0.978	-
Exp 2							
	Treatment	4	4	4	**<0.001**	**<0.001**	**<0.001**
	Variety	1	1	1	**0.013**	0.113	0.119
	Time	8	8	-	**<0.001**	**<0.001**	-
	Time × Treatment	32	32	-	**<0.001**	**<0.001**	-
	Time × Variety	8	8	-	0.502	0.526	-
	Time × Treatment × Variety	32	32	-	0.693	0.813	-
	CV (%)	5.2	6.5	4.3			
	Contrast *p* values						
	IRR vs. DTs	1	1	1	**<0.001**	**<0.001**	**<0.001**
	DTs vs. VGs	1	1	1	**<0.001**	**<0.001**	**<0.001**
	DT vs. DT2	1	1	1	**<0.001**	0.564	**<0.001**
	VGDT vs. VGDT2	1	1	1	**<0.001**	**<0.001**	**<0.001**
	Interaction contrast *p* values						
	Variety × Treatment	4	4	4	0.494	0.521	0.348
	Variety × IRR vs. DTs	1	1	1	0.348	0.885	0.804
	Variety × DT vs. VGDT	1	1	1	0.556	0.949	0.102
	Variety × DT vs. DT2	1	1	1	0.445	0.146	0.933
	Variety × VGDT vs. VGDT2	1	1	1	0.214	0.315	0.196
	Time × Treatment	32	32	-	**<0.001**	**<0.001**	-
	Time × IRR vs. DTs	8	8	-	**<0.001**	**<0.001**	-
	Time × DTs vs. VGDTs	8	8	-	**<0.001**	**<0.001**	-
	Time × DT vs. DT2	8	8	-	**<0.001**	0.343	-
	Time × VGDT vs. VGDT2	8	8	-	**<0.001**	**<0.001**	-
	Time × Variety	8	8	-	0.502	0.58	-
	Time × Variety × Treatment	32	32	-	0.693	0.881	-
	Time × Variety × IRR vs. DTs	8	8	-	0.631	0.745	-
	Time × Variety × DTs vs. VGDTs	8	8	-	0.741	0.347	-
	Time × Variety × DT vs. DT2	8	8	-	0.64	0.852	-
	Time × Variety × VGDT vs. VGDT2	8	8	-	0.265	0.806	-

**Table 6:** The yield of potato tubers from two varieties (Challenger and Russet Burbank) under different irrigation and Vapor Gard antitranspirant treatments across two experiments. Treatments included irrigated control (IRR), drought (DT), severe drought (DT2), and Vapor Gard application under moderate (VGDT) and severe drought (VGDT2). Values shown are means of replicate pots (*n* = 4 for Experiment 1, *n* = 5 for Experiment 2). Bold numbers indicate significant differences at *p* < 0.05. IRR = Irrigated; DT = Drought; DT2 = Severe Drought.

		Experiment 1	Experiment 2
Variety	Treatment	Weight (g)	Weight (g)
Challenger	IRR	1764	1662
	DT	651	1175
	DT2	610	997
	VGDT	1100	1590
	VGDT2	771	1117
Mean		979	1308
Russet Burbank	IRR	1394	1456
	DT	654	1025
	DT2	612	973
	VGDT	1080	1417
	VGDT2	709	1192
Mean		890	1213
Mean over varieties			
IRR		1579	1559
DT		653	1100
DT2		611	985
VGDT		1090	1504
VGDT2		740	1155
CV%		19.0	5.9
*p* values			
Treatment		**<0.001**	**<0.001**
Variety		0.376	0.073
Variety × Treatment		0.725	0.405
Contrast *p* values			
IRR vs. DTs		**0.008**	**0.004**
DTs vs. VGDTs		**0.01**	0.06
DT vs. DT2		**0.005**	**<0.001**
VGDT vs. VGDT2		**<0.001**	**<0.001**
Interaction contrast *p* values			
Variety × IRR vs. DTs		0.645	0.604
Variety × DTs vs. VGDTs		0.358	0.782
Variety × DT vs. DT2		0.943	0.37
Variety × VGDT vs. VGDT2		0.334	0.095
SED	d.f		
Treatment	27 (Exp 1) 36 (Exp 2)	156.8	82.1
Variety		99.1	51.9
Variety × Treatment		221.7	116.0

**Table 7:** Number and percentage of potato tubers with physiological disorders (russeting, internal brown spot [IBS]/necrosis, and jelly end rot) in Experiment 1 for two varieties, Challenger and Russet Burbank, under different irrigation and antitranspirant treatments. Data are presented as counts (n) with corresponding percentages in parentheses. Treatments: IRR = irrigated control, DT = drought without Vapor Gard, VGDT = drought + Vapor Gard, DT2 = severe drought without antitranspirant, VGDT2 = severe drought + Vapor Gard. A total of 24 tubers per treatment were assessed.

Variety	Treatment	Russeting	Number of Disorder-Free Tubers
		Experiment 1	
Challenger	IRR	3 (13%)	21 (88%)
	DT	17 (71%)	4 (17%)
	DT2	14 (58%)	10 (42%)
	VGDT	8 (33%)	16 (67%)
	VGDT2	7 (29%)	15 (62%)
Mean		9.8 (41%)	13.2 (55%)
Russet Burbank	IRR	0 (0%)	18 (75%)
	DT	3 (13%)	8 (33%)
	DT2	6 (25%)	3 (13%)
	VGDT	2 (8%)	16 (67%)
	VGDT2	3 (13%)	14 (58%)
Mean		2.8 (12%)	11.8 (49%)
	Treatment	Jelly End Rot	Number of Disorder-Free Tubers
Russet Burbank	IRR	2 (8%)	18 (75%)
	DT	2 (8%)	8 (33%)
	DT2	3 (12%)	3 (13%)
	VGDT	2 (8%)	16 (67%)
	VGDT2	3 (13%)	14 (58%)
Mean		2.4 (10%)	11.8 (49%)
	Treatment	IBS/Necrosis	Number of Disorder-Free Tubers
Challenger	IRR	1 (4%)	21 (88%)
	DT	3 (13%)	4 (17%)
	DT2	0 (0%)	10 (42%)
	VGDT	0 (0%)	16 (67%)
	VGDT2	2 (8%)	15 (62%)
Mean		1.2 (5%)	13.2 (55%)
Russet Burbank	IRR	4 (17%)	18 (75%)
	DT	11 (46%)	8 (33%)
	DT2	12 (50%)	3 (13%)
	VGDT	4 (17%)	16 (67%)
	VGDT2	4 (17%)	14 (58%)
Mean		7.0 (29%)	11.8 (49%)

**Table 8:** *p* values from orthogonal comparisons of physiological disorders (russeting, internal brown spot [IBS]/necrosis, and jelly end rot) and disorder-free across two potato varieties (Challenger and Russet Burbank) under different irrigation and antitranspirant treatments in two experiments. Bold values indicate significant differences at *p* < 0.05. Where IRR = irrigated control, DT = drought without antitranspirant, VG = Vapor Gard antitranspirant, and “-” indicates where comparisons were not applicable, or data were unavailable.

Exp	Variety	Comparison	Russeting	IBS/Necrosis	Jelly End Rot	Disorder-Free
1	Challenger	IRR vs. DTs	**<0.001**	**<0.001**	-	**<0.001**
1	Challenger	DT vs. DT2	0.059	**0.026**	-	**0.012**
1	Challenger	DTs vs. VGDTs	**<0.001**	0.125	-	**<0.001**
1	Challenger	VGDT vs. VGDT2	0.459	0.258	-	0.387
1	Russet Burbank	IRR vs. DTs	**0.002**	**<0.001**	0.213	**<0.001**
1	Russet Burbank	DT vs. DT2	0.054	0.099	0.139	0.051
1	Russet Burbank	DTs vs. VGDTs	**0.03**	**<0.001**	0.301	**<0.001**
1	Russet Burbank	VGDT vs. VGDT2	0.311	0.437	0.311	0.284
2	Challenger	IRR vs. DTs	**<0.001**	**<0.001**	-	**<0.001**
2	Challenger	DT vs. DT2	**0.004**	**0.029**	-	**<0.001**
2	Challenger	DTs vs. VGDTs	**<0.001**	0.191	-	**<0.001**
2	Challenger	VGDT vs. VGDT2	<0.001	0.051	-	**<0.001**
2	Russet Burbank	IRR vs. DTs	**0.002**	**0.001**	**<0.001**	**0.002**
2	Russet Burbank	DT vs. DT2	0.17	0.164	**<0.001**	**<0.001**
2	Russet Burbank	DTs vs. VGDTs	**<0.001**	0.091	**<0.001**	**0.001**
2	Russet Burbank	VGDT vs. VGDT2	0.079	0.051	0.267	0.612

**Table 9:** Number and percentage of potato tubers with physiological disorders (russeting, internal brown spot [IBS]/necrosis, and jelly end rot) in Experiment 2 for two varieties, Challenger and Russet Burbank, under different irrigation and antitranspirant treatments. Data are presented as counts (n) with corresponding percentages in parentheses. Treatments: IRR = irrigated control, DT = drought without antitranspirant, VGDT = drought + Vapor Gard, DT2 = severe drought without antitranspirant, VGDT2 = severe drought + Vapor Gard. A total of 40 tubers per treatment were assessed.

Variety	Treatment	Russeting	Number of Disorder-Free Tubers
		Experiment 2	
Challenger	IRR	4 (10%)	36 (90%)
	DT	25 (62%)	13 (32%)
	DT2	33 (82%)	3 (8%)
	VGDT	5 (12%)	34 (85%)
	VGDT2	18 (45%)	20 (50%)
Mean		17.0 (42%)	21.2 (53%)
Russet Burbank	IRR	0 (0%)	38 (95%)
	DT	5 (12%)	11 (28%)
	DT2	5 (12%)	23 (58%)
	VGDT	3 (8%)	31 (78%)
	VGDT2	0 (0%)	28 (70%)
Mean		2.6 (6%)	26.2 (66%)
Russet Burbank	Treatment	Jelly End Rot	Number of Disorder-Free Tubers
	IRR	0 (0%)	38 (95%)
	DT	16 (40%)	11 (28%)
	DT2	3 (8%)	23 (58%)
	VGDT	1 (2%)	31 (78%)
	VGDT2	0 (0%)	28 (70%)
Mean		4.0 (10%)	26.2 (66%)
	Treatment	IBS/Necrosis	Number of Disorder-Free Tubers
Challenger	IRR	0 (0%)	36 (90%)
	DT	2 (5%)	13 (32%)
	DT2	4 (10%)	3 (8%)
	VGDT	1 (2%)	34 (85%)
	VGDT2	2 (5%)	20 (50%)
Mean		1.8 (4%)	21.2 (53%)
Russet Burbank	IRR	2 (5%)	38 (95%)
	DT	8 (20%)	11 (28%)
	DT2	9 (22%)	23 (58%)
	VGDT	5 (12%)	31 (78%)
	VGDT2	12 (30%)	28 (70%)
Mean		7.2 (18%)	26.2 (66%)

## Data Availability

The original contributions of this study are included in the article. Further inquiries may be directed to the corresponding authors.
